# A peptide derived from TIMP-3 inhibits multiple angiogenic growth factor receptors and tumour growth and inflammatory arthritis in mice

**DOI:** 10.1007/s10456-013-9389-y

**Published:** 2013-10-16

**Authors:** Yung-Yi Chen, Nicola J. Brown, Rita Jones, Claire E. Lewis, Ahmed H. Mujamammi, Munitta Muthana, Michael P. Seed, Michael D. Barker

**Affiliations:** 1 Department of Oncology, Medical School, University of Sheffield, Beech Hill Road, Sheffield, S10 2RX UK; 2 Department of Infection and Immunity, Medical School, University of Sheffield, Sheffield, S10 2RX UK; 3 William Harvey Research Institute, Bart’s and the London School of Medicine and Dentistry, Queen Mary College University of London, London, EC1M 6BQ UK; 4 Present Address: School of Immunity and Infection, Institute of Biomedical Research, University of Birmingham, Birmingham, B15 2TT UK; 5 Present Address: Medicines Research Group, Health, Sport and Bioscience, University of East London, London, E15 4LZ UK

**Keywords:** TIMP-3, VEGFR2, Receptor, Angiogenesis, Arthritis, Tumour

## Abstract

The binding of vascular endothelial growth factor (VEGF) to VEGF receptor-2 (VEGFR-2) on the surface of vascular endothelial cells stimulates many steps in the angiogenic pathway. Inhibition of this interaction is proving of value in moderating the neovascularization accompanying age-related macular degeneration and in the treatment of cancer. Tissue inhibitor of metalloproteinases-3 (TIMP-3) has been shown to be a natural VEGFR-2 specific antagonist—an activity that is independent of its ability to inhibit metalloproteinases. In this investigation we localize this activity to the C-terminal domain of the TIMP-3 molecule and characterize a short peptide, corresponding to part of this domain, that not only inhibits all three VEGF-family receptors, but also fibroblast growth factor and platelet-derived growth factor receptors. This multiple-receptor inhibition may explain why the peptide was also seen to be a powerful inhibitor of tumour growth and also a partial inhibitor of arthritic joint inflammation in vivo.

## Introduction

Aberrant angiogenesis is now widely accepted as a key player in a variety of pathological conditions, including cancer, rheumatoid arthritis and ocular neovascularization. In the case of malignant tumours, angiogenesis facilitates tumour growth and metastasis, and in arthritis, the formation of an inflamed pannus.

Angiogenesis is a complex phenomenon that involves activation, migration and proliferation of endothelial cells, smooth muscle cells and pericytes. Various pro-angiogenic factors are involved in this process including epidermal growth factor (EGF), basic fibroblast growth factor (bFGF), platelet derived growth factor (PDGF) and the vascular endothelial growth factor (VEGF) family—VEGF-A being considered the predominant effector of angiogenesis. Vascular endothelial cells express the VEGF receptors VEGFR-1 and VEGFR-2, but only VEGFR-2 appears to mediate the pro-angiogenic effects of VEGF-A [1].

The growth of new blood vessels also requires breakdown of the surrounding extracellular matrix (ECM). The principal enzymes involved in this process are the matrix metalloproteinases (MMPs). Moreover MMPs also release ECM-bound angiogenic growth factors like VEGF-A, expose pro-angiogenic integrin binding sites in the ECM, and generate pro-migratory ECM derived fragments [2]. The activity of MMPs in tissues is tightly regulated by their endogenous inhibitors, the tissue inhibitors of metalloproteinases (TIMPs), and over-expression of these molecules in animal models inhibits tumour growth and metastasis [3]. However a completely novel anti-angiogenic activity has also been ascribed to one of the four TIMP family members, TIMP-3. TIMP-3 has been shown to specifically bind to, and inhibit, VEGFR-2 [4].

The initial aim of this investigation was to begin to map the site(s) on TIMP-3 involved in its interaction with VEGFR-2. We show that this involves the C-terminal domain of the TIMP-3 molecule. Interestingly, a short 16 amino acid peptide sequence, that forms the truncated C-terminus of a mutant form of TIMP-3, E139X, known to give rise to the degenerative retinal disease, Sorsby’s fundus dystrophy [5], also inhibited VEGF receptor-mediated signalling by endothelial cells (EC) in vitro. Additionally, unlike the normal TIMP-3 molecule, the peptide also moderated their responses to bFGF and PDGF in vitro. Finally, we show that this TIMP-3 peptide has potent inhibitory effects on murine tumour angiogenesis and growth as well as inflammatory arthritis in vivo.

## Materials and methods

### Proteins/peptides

Peptides corresponding to amino acids: Lys^123^–Asn^138^ (KIKSCYYLPCFVTSKN) of the C-terminal domain and Gly^69^–Arg^84^ (GLKLEVNKYQYLLTGR) from the N-terminal domain of TIMP-3 were synthesized by Sheaf Innovations Ltd., Sheffield, UK, and termed p700 and p323 respectively. Oxidation of the disulfide bond of p700 was confirmed by mass spectrometry. A 16 amino acid biologically inert control peptide, fibrinopeptide A (FPA) [6] was purchased from Bachem Distribution Services GmbH, Hegenheimer, Germany.

### Recombinant human TIMP-3 purification

WT-TIMP-3 or N-TIMP-3 cDNA, already bearing a 3′ 6× His tag and stop codon, were sub-cloned into the pIB/V5-His-TOPO vector and used to transfect High Five insect cells (Invitrogen, UK). Clones expressing the highest levels of recombinant protein were grown in suspension culture and the conditioned media collected and concentrated by ultrafiltration. Recombinant proteins were then partially purified on heparin-agarose columns (Sigma-Aldrich, UK), eluting with 0.5 M NaCl before a final purification on Ni^2+^-NTA-Agarose (Qiagen, UK), eluting with PBS containing 60 mM l-histidine and 0.025 % Brij-35 (pH 7.4). The purity of the eluted proteins was confirmed by SDS-PAGE followed by silver-staining and activity confirmed by reverse-zymography as previously described [7].

### Solid phase binding assay

Ninety six-well plates were coated with the extracellular domain of recombinant human VEGFR-2 fused to the Fc domain of human IgG1 (rhVEGFR-2, R&D Systems, UK) by overnight incubation followed by blocking in 5 % bovine serum albumin/PBS. Recombinant human VEGF_165_ (R&D Systems, UK), TIMP-3, N-TIMP-3 or peptides derived from TIMP-3 (p323 or p700) were titrated across the plates and then biotinylated-VEGF_165_ added to each well (2.4 nM final concentration) followed by incubation for 1 h at room temperature. After washing, the biotinylated-VEGF_165_ was detected with avidin-horse-radish peroxidase and OPD substrate (Sigma-Aldrich, UK) followed by spectrophotometry.

### Cells

Human dermal microvascular endothelial cells **(**HuDMEC), human umbilical vein endothelial cells (HUVEC), primary human synovial cells (SC), murine mammary tumour cells (4T1), and adult human lymphatic endothelial cells (LEC) were used in this study. HUVEC were maintained in Endothelial Cell Growth Medium MV, supplemented with 0.4 % (^v^/_v_) endothelial cell growth supplement/heparin (ECGS/H), 2 % (^v^/_v_) foetal calf serum (FCS), 0.1 ng/ml EGF, 1 μg/ml hydrocortisone, and 1 ng/ml bFGF. HuDMEC and LEC were maintained in Endothelial Cell Growth Medium MV2, supplemented with 0.4 % (^v^/_v_) ECGS/H, 5 % (^v^/_v_) FCS, 10 ng/ml EGF and 1 μg/ml hydrocortisone. All the primary human endothelial cells (passages 2–6 were used) and growth media were obtained from PromoCell (PromoCell, GmbH, Germany). 4T1 cells (ATCC, LGC Standards, UK) were maintained in RPMI-1640 medium (Lonza, UK) supplemented with 10 % (^v^/_v_) FCS and 2 mM l-glutamine. SC, isolated from rheumatoid arthritis patient biopsies, were a kind gift of Prof A. G. Wilson, University of Sheffield and cultured in DMEM (Lonza, UK) supplemented with 10 % (^v^/_v_) FCS and 2 mM l-glutamine. All primary cells or cell lines were maintained at 37 °C in a humidified 5 % CO_2_ atmosphere.

### Cell proliferation assay

Human dermal microvascular endothelial cells were serum-starved followed by incubating with p700 peptide for 1 h and then 0.5 nM of VEGF_165_ for 24 h at 37 °C. Cell proliferation was determined using a BrdU cell proliferation assay kit (Merck Chemicals Ltd, Nottingham, UK).

### Cell migration assay

The ability of the p700 peptide to inhibit VEGF_165_-induced EC migration was determined using a multi-well Boyden chamber (Neuro Probe, Gaithersburg, USA). Briefly, serum-starved HuDMEC were treated with p700 for 1 h, washed and plated in the upper chamber in EBM-2 medium containing 1 % FCS on an 8 μm fibronectin pre-coated polycarbonate filter (Neuro Probe, Gaithersburg, USA). The cells were then left to migrate across the filter for 4.5 h at 37 °C with media containing 0.5 nM VEGF_165_ in the presence and absence of inhibitors in the lower chamber. After the incubation, non-migrating cells on the upper side of the membrane were scraped off and the migrated cells were stained with Hema Gurr^®^ rapid staining kit (Merck Chemicals Ltd, Nottingham, UK). Migrated cells were counted using a light microscope at 160× magnification in three random fields per well of three separate wells.

### Tube formation assay

Serum-starved HuDMEC were seeded onto Matrigel™ matrix pre-coated 96 well plates (BD Biosciences, Cowley, Oxford, UK) at a density of 1 × 10^4^ cells/well in 100 μl EBM-2 medium. Peptides were then added into each corresponding well and incubated for 1 h at 37 °C, followed by stimulated with growth factors, 0.5 nM VEGF_165_, 1 nM PDGF-BB or 1.6 nM bFGF, to induce tubule development. The tubules formed were examined 6 h after stimulation under a low power (40×) light microscope. Images were captured and analysed by measuring the average tubule length using ImageJ software (National Institutes of Health, USA).

### Immunoblotting

Transfected HUVEC or HuDMEC pre-treated with 1 μM test peptides for 1 h, were stimulated with 0.5 nM of VEGF_165_ for 2, 5 or 10 min. Cells were lysed and phosphorylated proteins detected by western blotting using phospho-specific antibodies to either VEGFR-2, Erk1/2 or PI_3_K (New England BioLabs, UK). Band intensities were quantified using Bio-Rad Quantity One Analysis software and normalized using U, un-stimulated control, as 0 %; and VEGF_165_, positive control, as 100 % of protein phosphorylation.

### Phosphorylation RTK assays

Serum-deprived HuDMEC were pre-treated with test peptides for 1 h and then stimulated with growth factors (0.5 nM VEGF_165_, 1.6 nM bFGF, 1 nM PDGF-BB, 1.6 nM hepatocyte growth factor, 100 ng/ml EGF or 100 ng/ml insulin-like growth factor I) for 5 min. Cells were then lysed and phosphorylated proteins detected using (a) a receptor tyrosine kinase array (Human Phospho-Receptor Tyrosine Kinase Array Kit, R&D Systems Ltd.) or (b) sandwich ELISA (Duo-sets, R&D Systems Ltd.) according to the manufacturer’s instructions.

### Syngeneic mammary tumour model (4T1)

10^6^ 4T1 cells were subcutaneously (*s.c.*) implanted into the right flank of 5–6 weeks old female BALB/c mice (Charles River Laboratories, Margate, UK) and grown until the tumour size reached 100–350 mm^3^. The tumour sizes were measured using callipers as described previously [8]. Once the size of the tumours reached 100–350 mm^3^, mice were randomized into control and treatment groups and injected with either vehicle alone (PBS), 0.025, 0.25 or 2.5 mg/kg of p700 peptide in PBS, either directly into the tumour (*i.t.*) once per week for 2 doses; or intravenously (*i.v.*) every other day for 5 doses. At the end of the experiment, animals were culled and tumours and normal tissues, including lung and liver, excised and fixed with 10 % neutral buffered formalin for subsequent analysis, which included general histology and quantitative measurement of microvascular density (MVD) and necrosis.

### Histological analysis of 4T1 tumours: microvessel density and tumour necrosis

For MVD measurement, rabbit-anti-mouse CD31 (AbD Serotech, Kidlington, Oxford, UK) antibody was used and sections were stained according to standard immunohistological staining protocols for formalin-fixed tissue sections. Microvessel density was estimated by measuring the percentage of CD31 positive staining in the tumour sections (15 areas per tumour slide, 7 tumour slides per group) using AnalySIS^^^D image analysis software (Olympus, UK). For the necrotic area inside the tumour, tumour slides were stained with H&E. Images were measured at 200× magnification, photographed (15 areas per tumour slide, 7 tumour slides per group) and analysed by AnalySIS^^^D image analysis software (Olympus, UK).

### Analysis of 4T1 metastases

Normal tissues including lung and liver were stained with H&E to study tumour metastasis. Total number of metastases per mouse was determined as the number of metastatic foci on five sections for each animal (*n* = 7). Incidence of metastasis was calculated as the percentage of mice with one or more metastatic nodules in the tissues. Metastasis severity was scored as follows [9]: minimal (score 0 = no metastatic nodule), minimal (score 1 = ≤ 4 metastatic nodules), medium (score 2 = 5–7 metastatic nodules) or extensive involvement (score 3 = ≥ 8 metastatic nodules). Each nodule contained ≥5 nuclei.

### In vitro synovial cell invasion assay

Synovial cell invasion was determined using a GFR-Matrigel™ invasion assay kit (BD Biosciences, USA). Serum-starved SC were treated with peptide for 1 h, followed by stimulation with 100 ng/ml PDGF-BB at 37 °C for 22 h. After incubation, non-invading cells on the upper side of the chamber were removed and the invaded cells on the lower side of the membrane were fixed with methanol and stained with Hema Gurr^®^ rapid staining solution (Merck Chemicals, Ltd.). Cells were counted using a light microscope at 160× magnification in 3 random fields per well of 3 separate wells.

### Collagen-induced-arthritis mouse (CIA) model

The CIA mouse was used to evaluate the effect of p700 in rheumatoid arthritis. Briefly, male DBA-1 mice were sensitized to bovine collagen type II in Freund’s complete adjuvant, and synchronized with a boost of collagen in incomplete adjuvant on day 21. Mice were lightly anesthetized with halothane. The base of the tail was shaved and 100 μl collagen II/FCA emulsion (0.1 mg *M.tb.,* H37RA, Sigma/100 μL Freund’s incomplete adjuvant, Difco; Final concentration 100 μg collagen II/100 μL FIA) was injected intradermally to the left hand side of this site. 21 days after initial sensitization, collagen II was dissolved in acetic acid as above, emulsified 1:1 in Freund’s incomplete adjuvant and 100 μL injected into the base of the tail on the right hand side of the tail base. Mice were then intravenously treated with vehicle control (PBS) or p700 peptide (2.5 mg/kg) for 7 doses, *i.v.* every 2 days into alternating sides of tail veins from day prior to boost (day 20) to day 35.

The development of the arthritis was assessed by blind observation of ‘clinical joint score’ (i.e. arthritis scores) [10]. Mice were individually marked and examined from the time of the day of boost (day 21). Every inflamed main digit scored one, inflammation of the front paw scored one, inflammation of the hind paw scored one, and involvement of the ankle scored one. Thus a maximal score for each animal was 22. Animals were also assessed quantitatively for hind paw inflammation through volumetric measurement by plethysmometry (Ugo Basille Srl, Italy) and expressed as mL change from day 21 boost baseline. Mice with arthritis development pre boost were omitted from the final analysis. Results were also expressed as area under the curve (AUC) from day 21.

### In silico modelling of the TIMP3/VEGFR2 interaction

In order to attempt to assess how TIMP3 and the p700 peptide might bind to and inhibit VEGFR2, in silico modelling of the interaction was performed. While a crystal structure for the whole of TIMP-3 is not yet available, the structures of full length TIMP1 and TIMP2 and the N-domain of TIMP3 have been solved at high resolution [11–14] enabling a model of the full length TIMP3 protein to be created using the Phyr2 Server [15] with high degree of confidence (94 % modeled at >90 % confidence). This was uploaded to the ZDOCK protein docking server [16] together with the crystal structure of the D23 immunoglobulin-homology domains of VEGFR2, that comprises the VEGFA binding site [17]. From the predicted structures, the residues present in both molecules that lie within 4 Å of one another were determined using PyMOL software.

## Results

### Inhibition of ligand binding to VEGFR-2

**Fig. 1:**
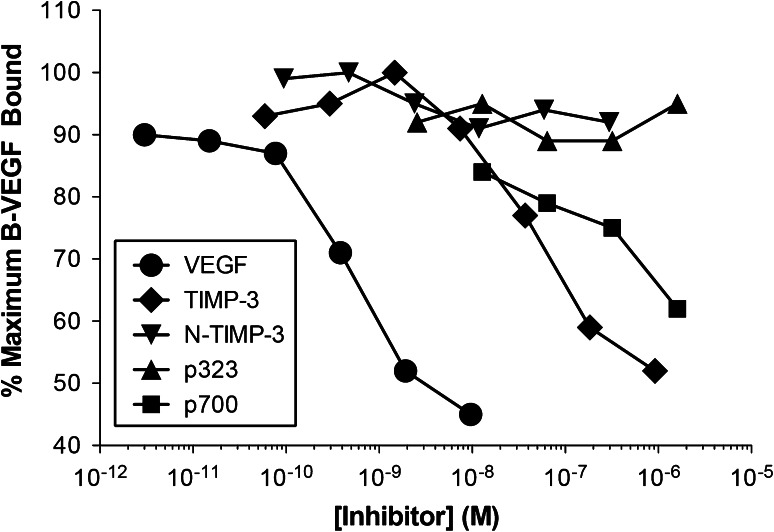
The binding of biotinylated-VEGF_165_ (B-VEGF) to rhVEGFR-2 following pre-incubation with either unlabelled VEGF_165_ (VEGF_165_), WT-TIMP-3 (WT-T3), N-TIMP-3 (N-T3), p700 peptide (p700) or p323 peptide (p323) relative to the binding of B-VEGF alone

We had previously found that HUVEC cells transfected with cDNA corresponding to full length TIMP3 and a highly truncated Sorsby’s fundus dystrophy mutated form of TIMP3, E139X, showed reduced VEGFR2 and Erk1/2 phosphorylation in response to VEGF, whereas the same cells transfected with the N-terminal domain showed no reduction in these responses (unpublished observation). The only difference between the N-terminal domain of TIMP-3 and the E139X mutant is a sequence of 16 amino acids (Lys^123^–Asn^138^). This implicated a role for this domain in VEGFR-2 binding. In order to directly test this hypothesis a peptide corresponding to this sequence (p700), together with other TIMP-3 derived sequences, were tested for their ability to inhibit the binding of biotinylated-VEGF_165_ to the extracellular domain of recombinant human VEGFR-2 (Fig. 1). This showed that full length TIMP-3 and p700, but not N-TIMP-3 or p323, a 16 amino acid peptide sequence from the N-terminal domain, were able to inhibit the binding of VEGF_165_ to rhVEGFR-2. Complete inhibition of B-VEGF_165_ was not observed for any of the proteins as increasing inhibitor concentrations much above those shown led to non-specific binding of B-VEGF_165_ to the plate, possibly due to the basicity of all these peptides.

In order to confirm this effect on the endogenous VEGFR-2 receptor, a series of in vitro angiogenesis assays were performed using human dermal microvascular endothelial cells (HuDMEC).

### Inhibition of VEGF_165_-induced functional responses of HuDMEC

#### Cell proliferation and migration

**Fig. 2:**
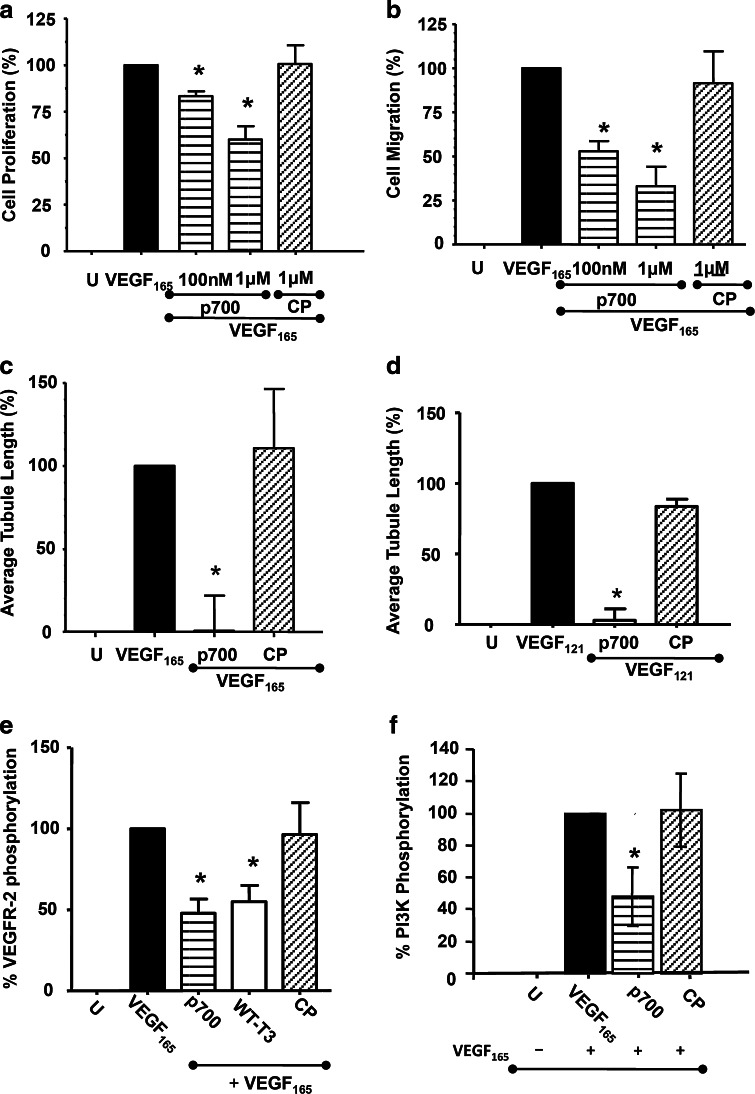
p700 inhibits VEGF-mediated responses in HuDMEC. p700 showed a dose responsive inhibition of **a** VEGF_165_-induced HuDMEC proliferation, **b** cell migration and (at 1 μM) almost totally abolished both **c** VEGF_165_- and **d** VEGF_121_-induced tubule formation. Mean ± SEM, n = 4, *p < 0.05 compared to positive control, Mann–Whitney *U* test). Similarly p700 inhibited VEGF_165_-induced phosphorylation of **e**VEGFR-2 itself and **f** downstream kinase PI_3_K (means ± SEM, n = 3. *p < 0.05 *w.r.t.* positive control, Mann–Whitney *U* test). All data were normalized using U, untreated cells, as 0 % and VEGF only-treated cells as 100 % activity. In all cases the control peptide (CP) had no significant effect

p700 showed a dose responsive inhibition of VEGF_165_-induced cell proliferation (to a maximum inhibition of 40 %) and migration (maximum inhibition, 67 %) up to a concentration of 1 μM (Fig. 2a, b). Concentrations of the control peptide of 10 μM and above showed some toxicity to HuDMEC (as determined by propidium iodide staining—data not shown) so that the optimum dose of peptides used in subsequent experiments was 1 μM.

#### Tubule formation

Exposure to 1 μM p700 virtually abolished VEGF_165_-induced HuDMEC tube formation (>99 % inhibition) (Fig. 2c). The binding of VEGF_165_ to VEGFR-2 has been shown to be augmented by heparan sulfate proteoglycans (HSPG) or exogenous heparin that interacts with both molecules [18, 19]. TIMP-3 also binds to sulfated glycosaminoglycans [20], and while this does not seem to be responsible for its ability to inhibit VEGFR-2 in that TIMP-3 also inhibits responses to VEGF_121_ (which lacks a heparin binding site) [4], there remained a possibility that p700, being highly basic (calculated pI 9.74), could bind to heparin, potentially affecting the ability of VEGF_165_ to bind to VEGFR-2. However p700 was similarly potent (>97 % inhibition) at inhibiting tubule formation in response to VEGF_121_ (Fig. 2d). Addition of the peptide in the absence of VEGF had no effect on tubule formation, relative to untreated cells (data not shown).

#### VEGFR-2-mediated signalling

1μM p700 significantly inhibited VEGF_165_-induced VEGFR-2 phosphorylation by approximately 50 % and this was comparable to the inhibition by the same concentration of rhTIMP-3 (45 % inhibition) (Fig. 2e). Similarly, 1 μM p700 significantly inhibited VEGF_165_-induced phosphorylation of downstream kinase PI_3_K by approximately 50 % (Fig. 2f).

### Inhibition of other growth factor receptors

**Fig. 3:**
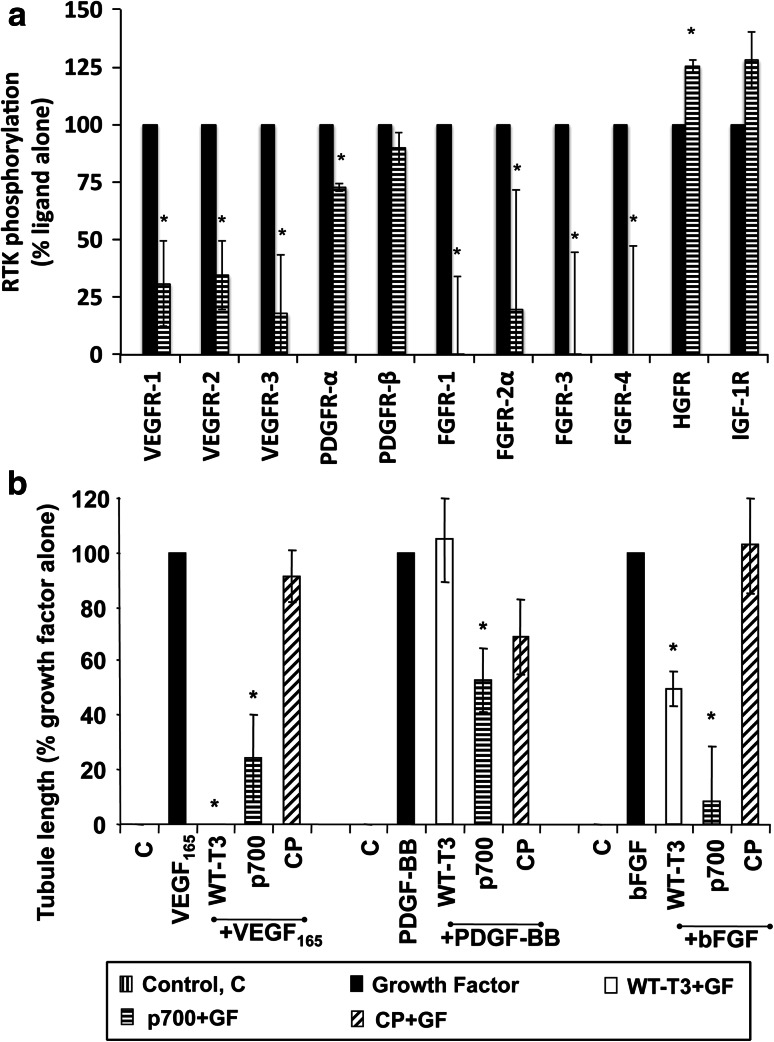
p700 peptide inhibits phosphorylation of multiple tyrosine kinase receptors. **a** p700 peptide significantly inhibited VEGFR-1, -2 and -3, PDGFR-α, FGFR-1, -2α, -3 and -4 phosphorylation in response to their respective ligands. **b** p700 inhibited VEGF, bFGF and PDGF-BB stimulated tubule formation in vitro. Control peptide (CP), growth factor (GF), WT-TIMP-3 (WT-T3). Means ± SEM, n = 3. *p < 0.05 *w.r.t* positive control (Mann–Whitney *U* test)

A preliminary screen using a phospho-receptor tyrosine kinase array indicated p700 inhibition of additional VEGF, FGF and PDGF receptors but not the receptors for EGF, hepatocyte growth factor (HGFR/Met) or insulin-like growth factor receptors (data not shown). These data were then confirmed using a sandwich ELISA to show significant inhibition of VEGFR-1, -2 and -3, FGFR-1, -2α, -3 and -4 and PDGFR-α with no inhibition of HGFR or insulin-like growth factor 1 receptor (Fig. 3a). Additionally p700 inhibited tubule formation in response to VEGF_165_, bFGF and PDGF-BB (Fig. 3b).

### Effect of p700 on tumour growth

**Fig. 4:**
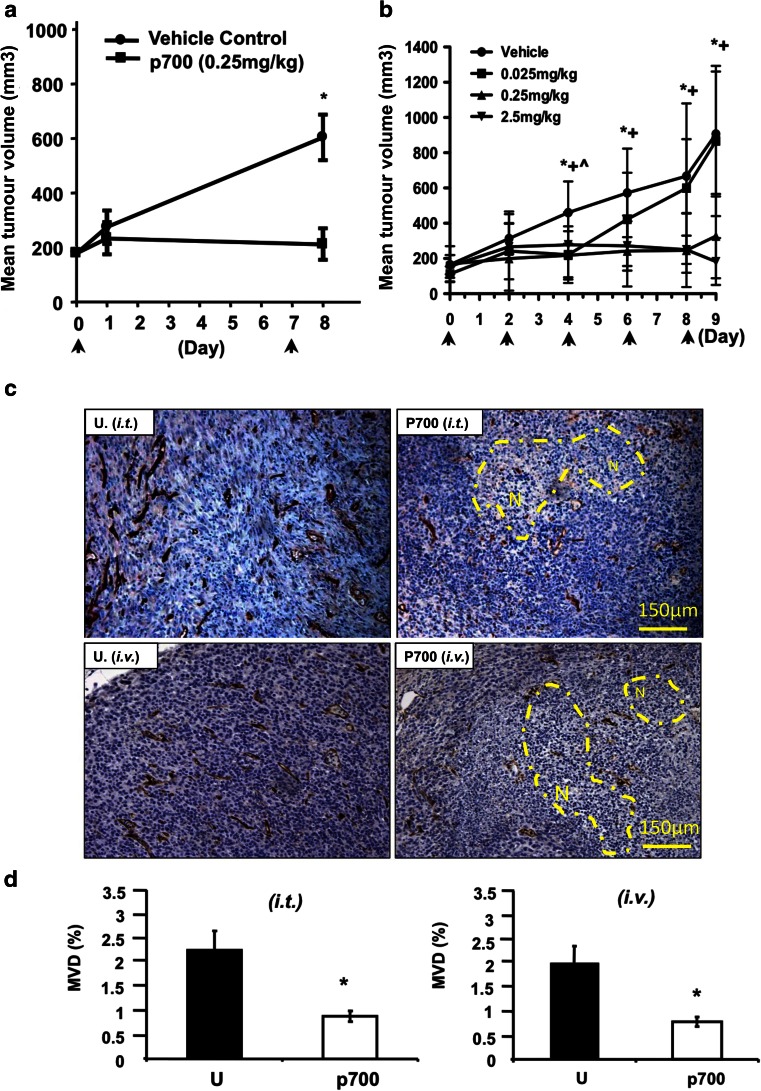
p700 reduces the growth of 4T1 mammary adenocarcinoma in mice and inhibits tumour angiogenesis. Animals were injected with either PBS (vehicle control) or PBS containing 0.025, 0.25 or 2.5 mg/kg p700 peptide—either **a** directly into the tumour (*i.t.*) or **b** intravenously (*i.v.*)—at the times indicated by the arrows, and tumour size measured using callipers so that the average tumour volume could be calculated. Data are means ± SEMs, n = 7. At the end of the procedure, the differences in mean tumour volume between vehicle control and p700 treated groups were significant (*i.t. route: *p* < 0.0025 and *i.v.* route: ^0.025 mg/kg treated group, *p* < 0.05; *0.25 mg/kg treated group, *p* < 0.02; +2.5 mg/kg treated group, *p* < 0.05; Mann–Whitney *U* test). **c** Tumour vasculature. Tumours from control animals (U), or p700-treated (p700), were immunostained for CD31 (*brown*) and photographed using an Olympus light microscope. *Bar* 150 μm. *N* necrotic area. Microvascular density (MVD) was then quantified **d** using AnalySIS^D image analysis software. The results show p700 significantly inhibits microvascular density in both *i.v.* and *i.t.* treatment groups. Data are means ± SEMs, n = 7 tumours/group. *p < 0.05 *w.r.t.* vehicle control group (Mann–Whitney *U* test)

The effect of p700 on tumour growth in vivo was demonstrated in a syngeneic breast tumour model (4T1 cells) in BALB/c mice. Tumours in both vehicle control groups (intravenous and intra-tumour injection sites) grew steadily and reached the maximum size allowable by day 24, when the animals were sacrificed. By contrast a weekly intra-tumour (*i.t.*) dose of 0.25 mg/kg peptide or intravenous (*i.v.*) dose every 2 days of 0.25 mg/kg or above almost totally abolished tumour growth (Fig. 4a, b).

### Effect of p700 on tumour histology

Anti-CD31 staining of the tumour sections revealed a significant (approximately 50 %) reduction in microvascular density in both treatment groups compared to that seen in the control groups (Fig. 4c, d). This may, at least in part, account for the observed inhibition in tumour growth. While there was an indication of increased necrosis and decreased metastases in the treated group, this failed to reach significance.

### In vitro effects of p700 on synovial cell invasion

**Fig. 5:**
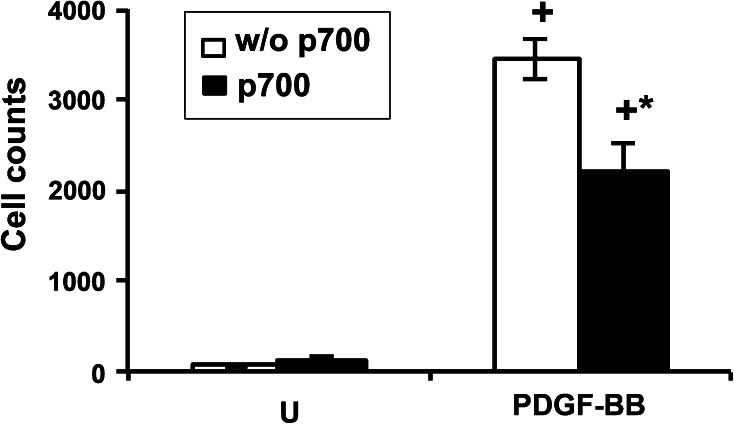
p700 inhibits PDGF-BB induced synovial cell invasion. U: untreated cells with (*solid bar*) or without (*open bar*) p700; PDGF-BB: Cells pre-treated with or without p700 and activated with PDGF-BB. Pooled data are shown as means ± SEM, n = 3. +*p* < 0.05 wrt U; *p < 0.05 wrt PDGF alone (Mann–Whitney *U* test)

The growth factor receptor inhibitory profile of p700 indicated it may also have an effect on the invasive phenotype of rheumatoid arthritis synovial cells, which lack VEGFR2 [21], but respond to PDGF and fibroblast growth factor (FGF). While synovial cells failed to migrate through Matrigel™ in response to bFGF (data not shown), they showed marked invasion in response to PDGF-BB and this response was significantly inhibited (by approximately 40 %) in the presence of p700 (Fig. 5).

### Effect of p700 on murine collagen-induced arthritis

**Fig. 6:**
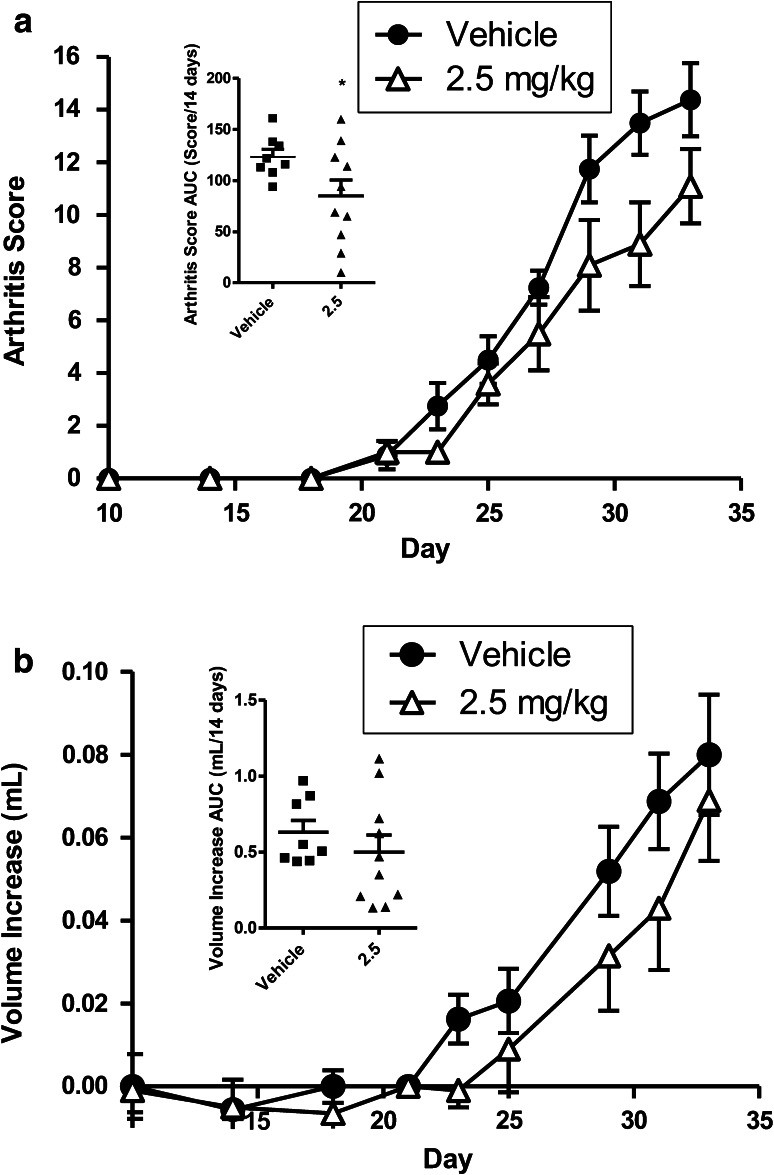
The inhibition of collagen-induced arthritis by p700. Mice with CIA were dosed with vehicle or p700 at 2.5 mg/kg. **a** Arthritis score (*p* < 0.001; 2-way ANOVA), and area under curve (*inset*, *p* < 0.05) relative to vehicle alone. **b** Hind paw volume (*p* < 0.05; 2-way ANOVA), and area under curve (*inset*, NS). Means ± SEM (Vehicle n = 8, p700 n = 10—points on AUC graphs are individual animals)

In order to test whether the effect of p700 on synovial cell invasion would translate into a therapeutic effect in vivo, the peptide was tested in a mouse collagen-induced arthritis (CIA) model. Injections of 2.5 mg/kg p700 every 2 days significantly inhibited CIA progression (Fig. 6a, b). Both the clinical presentation of disease as well as hind paw inflammation were reduced.

### In silico modelling of the TIMP3/VEGFR2 interaction

**Fig. 7:**
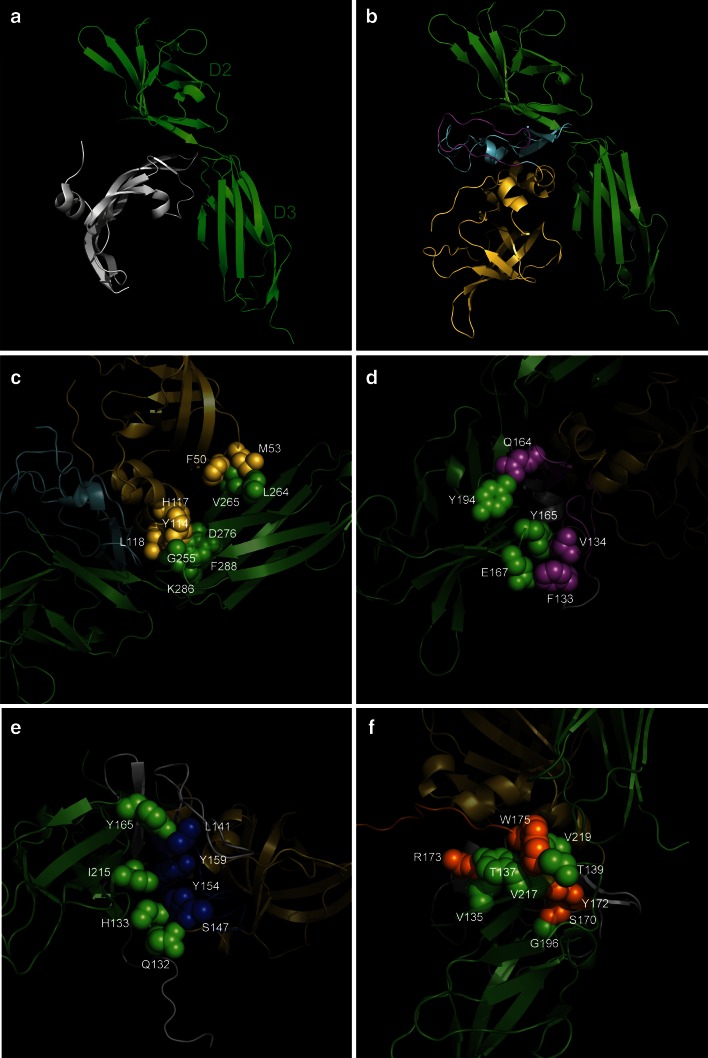
ZDOC modelling of potential TIMP-3/VEGFR2 interactions. Residues shown as spheres on TIMP-3 and VEGFR2 lie within 4 Å of one another. Domains D23 of VEGFR2 are shown in *green* throughout. **a** VEGFA (*grey*) bound to VEGFR2 D23 [17]. **b** ZDOC model of TIMP-3 (*yellow* N-domain; *pale blue* C-domain; *purple* p700 residues) bound to VEGFR2 D23. **c** TIMP-3 N-domain (*yellow*) binding residues. **d** Loop 4 TIMP-3 (*purple*) binding residues. **e** Loop 6 TIMP-3 (*blue*) binding residues. **f** C-tail peptide (*orange*) binding residues

The top 5 predicted TIMP-3/VEGFR2 interactions from the ZDOC server were examined. Figure 7a shows the known structure [17] of VEGFA bound to domains D23 of VEGFR2 and Fig. 7b, the highest scoring model of TIMP-3 bound to the same receptor domains, with the p700 sequence highlighted. Figure 7c–f show residues from the N-domain, loop 4, loop 6 and the C-terminal tail of TIMP-3 respectively, and the corresponding VEGFR2 residues, that lie within 4 Å of one another, highlighted in space filling mode using PyMOL.

## Discussion

Initially it was assumed that the anti-angiogenic activities of TIMPs were due to their ability to inhibit MMPs. However TIMP-2 and TIMP-3 have since been shown to exhibit anti-angiogenic activities that are independent of MMP inhibition [4, 22]. For TIMP-2 this appears to be due to its ability to bind α3β1 integrin [23] whereas for TIMP-3 it is due to its ability to bind to and inhibit VEGFR-2 [4]. The ability of TIMP-3 to inhibit VEGFR-2 mediated angiogenesis may play a critical role in the eye, regulating vascularization of the retina [24, 25]. Indeed specific mutations in TIMP-3 result in the autosomal dominant degenerative disease of the macula, Sorsby’s fundus dystrophy (SFD), frequently characterized by abnormal retinal vascularization [26].

While the ability of TIMPs to inhibit MMPs lies within their N-terminal domain, a number of activities that are specific to individual family members, such as pro-MMP binding, have been shown to reside in the C-terminal domain [27]. The data presented here indicate that this domain is also largely responsible for VEGFR-2 inhibition. This finding supports the observation that full length and C-terminal domain TIMP-3, but not N-TIMP-3 are able to inhibit aortic endothelial cell sprouting in mice [24]. Moreover the fact that p700 was able to mimic both the anti-angiogenic effects of TIMP-3, and its ability to inhibit VEGF binding to VEGFR-2, strongly implicates this proximal region of the C-terminus as the region that interacts with the VEGF binding site of the receptor.

The fact that TIMP-3 and p700 were less potent than unlabelled VEGF_165_ in competing with B-VEGF_165_ for receptor binding may simply reflect the fact that both the recombinant VEGFR-2 and VEGF_165_ are dimers, potentially resulting in a significant avidity effect, whereas TIMP-3 is monomeric. Indeed it has been observed that monovalent VEGFR-2 has an approximately 100-fold lower affinity for VEGF_165_ than the dimer [28].

TIMP-3 appears to bind VEGFR-2 specifically, with no inhibition of the closely related VEGFR-1, FGFR1 or PDGFR-β receptors [4]. In contrast, the p700 sequence also significantly inhibited the phosphorylation of VEGFR-1, VEGFR-3, PDGFR-α and FGFR-1, -2, -3 and -4 by their respective ligands, as well as VEGF-A PDGF-BB and bFGF-induced tubule formation. In the latter experiment some inhibition of bFGF-induced tubule formation was also seen with TIMP-3, which is in keeping with earlier reports demonstrating TIMP-3 inhibition of both VEGF-A and FGF-mediated angiogenesis in vitro and in vivo [29]. However a more recent report from the same laboratory showed that TIMP-3 did not inhibit bFGF or PDGF-BB induced proliferation of FGFR1 or PDGFR-β-transfected porcine aortic endothelial cells respectively, or PDGF-BB induced binding or phosphorylation of PDGFR-β in the same cells [4]. A likely explanation for this apparent discrepancy is the previously observed synergy between VEGF and FGF, whereby a significant proportion of the angiogenic effect of FGF on endothelial cells is dependent on the activity of autocrine and paracrine VEGF induced by FGF [30]. Clearly, however, this would not explain the inhibition of phosphorylation of FGF or PDGF receptors observed in our studies. Moreover, the fact that p700 potently inhibits PDGF-induced invasion of synovial cells, which lack VEGF tyrosine kinase receptors [21], confirms a direct effect on PDGFR.

The pattern of inhibition of p700 for additional tyrosine kinase receptors is in keeping with their structural conservation [31], as all have extracellular domains comprising immunoglobulin-homology repeats, with domains 2 and 3 (D23) playing major or exclusive roles in ligand binding. In contrast EGFR, HGFR or IGF-1R, which were not inhibited by p700, lack this domain structure. Indeed the in silico modelling using ZDOC indicated that TIMP-3 potentially occupies the D23 interface in a very similar manner to the ligand. Closer examination of this model showed 2 residues found in the p700 sequence, Phe133 and Val134 potentially in close contact with the ligand binding site. Although our data, and those recently published by Qi et al.[32], excluded the amino domain of TIMP-3 in VEGFR2 inhibition, some potential contact residues were also found there. This does not necessarily discredit the model, as it is possible these residues help stabilise the interaction but are insufficient to allow binding of N-TIMP-3 alone. However the majority of residues of TIMP-3 in contact with VEGFR2 in the model lie in the carboxyl domain, with both loop 6 and the COOH-tail sequence showing several sites of direct interaction. This is supported by the fact that peptides corresponding to these domains are also potent inhibitors of VEGFR2 [32]. While these authors failed to show inhibition with a 10 amino acid peptide corresponding to loops 4 and 5, that peptide lacks some of the residues found in p700 shown in the model to lie in the ligand binding site. The fact that p700 is highly promiscuous compared to the natural ligands, or indeed TIMP-3 itself, is presumably due to the diminutive size of the p700 peptide potentially abrogating specificity constraints of those much larger molecules.

While VEGF-A is considered the major angiogenic activator for endothelial cells, other growth factors, including bFGF [30] and PDGF-BB [33], synergize with VEGF or are directly angiogenic. Moreover angiogenesis is also dependent on pericytes and smooth muscle cells that stabilize the new vessels, and these cells respond to other growth factors, including bFGF and PDGF-BB, rather than VEGF. The latter growth factors are also potent mitogens for tumour cells [34]. These molecules may also play important roles in rheumatoid arthritis with, for example, bFGF being a potent inducer of osteoclastogenesis [35] and PDGF-BB, together with transforming growth factor beta, greatly potentiating fibroblast-like synoviocytes’ response to inflammatory cytokines [36].

This wider inhibitory profile of p700 may account for the apparent potency of the molecule in preventing tumour growth in the 4T1 mouse model and, to a lesser extent, in modulating arthritis in the CIA mouse model. While there was no significant decrease in tumour metastasis in the 4T1 model, this is a highly invasive and aggressive tumour and earlier dosing may have been more effective. The fact that the efficacy of p700 in the CIA model was less dramatic than in the 4T1 tumour model is not surprising. Subclinical disease and angiogenesis already occurs by day 21, and the boosted model provides a robust immune response. In addition, the fibroblastic role in arthritic disease occurs in the later chronic phases of the disease. A more potent action may have been effected at a higher dose, or with daily dosing, however resources did not permit these additional regimes. Angiogenesis plays an important role in chronic inflammation [37], and its inhibition results in protection against cartilage erosion [38]. Therefore drugs targeting angiogenic/growth factor receptors may provide an additional treatment modality to those targeting the immune system alone. Interestingly, in the mouse K/BxN model of RA, only inhibition of VEGFR-1 and not VEGFR-2 inhibits disease [39]. VEGFR-1 is also expressed by monocytes/macrophages and osteoclast precursor cells [40] and it is possible that angiogenesis and tissue damage in the rheumatoid joint is largely mediated by infiltration with these cells which then secrete further angiogenic and catabolic factors, rather than by direct interaction with VEGFR-2 on endothelial cells.

The majority of anti-angiogenic therapies currently in clinical practice target the VEGF/VEGFR interaction and fall into two main categories. The first are antagonists that target VEGF itself, the major player being bevacizumab [41], a humanized monoclonal antibody. The second are small molecule inhibitors that target the tyrosine kinase domain of these receptors and include sorafenib and sunitinib [42]. The former have to be administered intravenously and only target VEGF-A. However VEGF-A may be replaced as an angiogenic factor by other growth factors as disease progresses, including VEGF-C and -D, which can activate VEGFR-2 after proteolytic cleavage [43], placental growth factor which specifically targets VEGFR-1 [44], bFGF and PDGF [45]. The tyrosine kinase inhibitors can be taken orally and will also inhibit responses to all VEGF family members. Current drugs also inhibit several tyrosine kinase receptor family members in addition to VEGFR-2, including some intracellular signalling molecules. While this may broaden their activity against other pro-angiogenic factors it may also increase their off target effects.

The data described herein not only implicates the proximal region of the carboxyl-terminal domain of TIMP-3 in binding and inhibiting VEGFR-2, it also demonstrates the feasibility of using a single drug to target multiple tyrosine kinase receptors via their extracellular ligand binding domain, rather than their tyrosine kinase domain. This may provide an additional mode of targeting this family of receptors that potentially combines the benefits of current anti-angiogenic therapies; having a broader inhibitory profile than antibodies, but without the potential increased toxicity of small molecule tyrosine kinase inhibitors that can affect both intracellular signalling molecules as well as receptor tyrosine kinases. Moreover, understanding the mechanism of binding of this peptide to these receptors could pave the way for the rational design of drugs that target very specific groups of tyrosine kinase receptors.
